# Randomized Controlled Study Comparing Use of Propofol Plus Fentanyl versus Midazolam Plus Fentanyl as Sedation in Diagnostic Endoscopy in Patients with Advanced Liver Disease

**DOI:** 10.1155/2017/8462756

**Published:** 2017-09-26

**Authors:** Sameh Abdelkhalik Ahmed, Amal Selim, Nehad Hawash, Ahmed Khaled Tawfik, Mohamed Yousef, Abdelrahman Kobtan, Rehab Badawi, Sally Elnawasany, Reham Abdelkader Elkhouly, Amr Shaaban Hanafy, Fatma H. Rizk, Loai Mansour, Sherief Abd-Elsalam

**Affiliations:** ^1^Department of Anesthesia, Tanta University, Tanta, Egypt; ^2^Internal Medicine Department, Tanta University, Tanta, Egypt; ^3^Tropical Medicine Department, Tanta University, Tanta, Egypt; ^4^Internal Medicine Department, Zagazig University, Zagazig, Egypt; ^5^Department of Physiology, Tanta University, Tanta, Egypt

## Abstract

**Objectives:**

We aimed to investigate the safety and efficacy of propofol plus fentanyl versus midazolam plus fentanyl as sedative for patients with advanced liver disease presented for gastrointestinal endoscopy.

**Methods:**

A total of 100 patients with liver cirrhosis referred for upper endoscopy were enrolled and divided equally in two groups, midazolam plus fentanyl group and propofol plus fentanyl group. All patients were subjected to history taking, estimation of level of sedation, endoscopist rating, and hemodynamic parameters including oxygen saturation, heart rate, mean arterial pressure, incidence of side effect as (bradycardia, hypotension, hypoxia, nausea and vomiting, cough, shivering, or diplopia), time needed for complete recovery, and time needed for discharge.

**Results:**

There was no statistical significant difference between the studied groups regarding age, sex, weight, Child–Pugh classification score, type and duration of endoscopic intervention, time needed for complete recovery, or time needed for discharge. Complication rates were similar in both groups except for mean arterial blood pressure which was significantly lower in group of patients receiving propofol and fentanyl (*P* = 0.001).

**Conclusion:**

The use of either propofol or midazolam in combination to fentanyl is effective in sedation of patients with advanced liver diseases presented for upper GIT endoscope. The trial is registered with ClinicalTrials.gov Identifier: NCT03063866.

## 1. Introduction

Gastrointestinal endoscopy is a common procedure in the patients with advanced liver disease. It requires variable level of sedation ranging from minimal sedation to standard general anesthesia aiming for alleviating pain, anxiety, tension, and awful memories of the procedure [1].

In conscious sedation, patients are capable of making purposeful responses to auditory and tactile clues, with maintenance of ventilatory and circulatory stability. Certainly, even as, in deep sedation, patients react only to painful stimuli, they regularly require airway support. At the level of general anesthesia, patients are unresponsive, and airway support is obligatory [2, 3].

The use of general anesthesia or deep sedation in patients with advanced liver disease carries an increased risk of perioperative morbidity and mortality, even with minor procedures. Thus, the use of conscious sedation for endoscopy in hepatic patients remains as the classical method [4, 5].

The level of sedation may require changing throughout the same procedure. Generally, diagnostic procedure usually can be managed by conscious sedation, while therapeutic interventions need more deep level of sedation [6, 7].

Propofol, the short acting intravenous anesthetic agent, is used to achieve a different level of sedation nowadays. Some authors recommend the use of propofol as a sedative agent in short procedure [8].

Midazolam, the short acting barbiturate, is a sedative drug that exerts its action through the effect on GABA receptor in the brain, thus relieving anxiety. It has sedative, amnesic, and anticonvulsant effect [9]. However, midazolam shows extensive hepatic metabolism that increases the risk of complications in patients with advanced liver disease especially with prolonged sedation [10].

Fentanyl, the short acting opioid, is commonly used as an additive to the sedative agents to improve the sedation level, analgesia, and amnesia and to allow the decrease in the used dose of the sedative [11].

The aim of this study is to compare the use of propofol plus fentanyl versus midazolam plus fentanyl as sedative for patients with advanced liver disease presented for gastrointestinal endoscopy. Our primary outcome is the safety and efficacy of the drugs.

## 2. Patients and Method

This randomized, double-blind prospective study was carried out on 100 adult patients admitted to Department of Gastroenterology, Tanta University Hospital, who presented for elective endoscopy.

The duration of study was 6 months starting immediately after obtaining ethical committee approval. An informed written consent was taken from each patient and the study was registered on clinicaltrials.gov (ClinicalTrials.gov Identifier: NCT03063866). All patients' data were confidential with secret codes and will be used for the current study only.

Any unexpected risk appearing during the course of the research was cleared to the participants and ethical committee on time and proper measures were taken to overcome or minimize these risks.

*Risk to the Patients*. Patients included in the study who had potential risk of airway obstruction were managed with adequate monitoring, anesthetist attendance, and available airway management devices.

There was adequate provisions to maintain privacy of participants and confidentiality of the data.

There were no conflicts of interest, or conflicts with religion, law, or social obligations.

The research was useful for society and carried no risk for environmental pollution.

Patients included in this study were aged 40–60 years old, with CTP score B or C presented for elective gastrointestinal endoscopy.

Patients were excluded from the study in the following cases: if they refused to participate in the study and patients with emergent condition like hematemesis, patients with any grade of hepatic encephalopathy, patients with hepatopulmonary syndrome, or patients with known or suspected hypersensitivity to the used medication.

All the maneuvers were performed in the operating theatre with attendance of anesthesiologist throughout the whole procedure for initiation and maintenance of patients sedation with monitoring of the patients^,^ vital parameters. After preoperative evaluation of the patients through history, examination, and investigations, patients were counseled, reassured, and, then, transported to the operating theatre. Then, intravenous 22 gauge peripheral cannula was inserted. The patient was monitored with three leads, electrocardiogram, pulse oximetry, and noninvasive blood pressure. Supplemental oxygen (3-4 L/min) was given via a nasal cannula in all cases during the procedure with administration of 10 ml/kg of lactated ringer solution by i.v. infusion.

The patients were randomly allocated (using closed envelop method) into two groups, 50 patients in each group.

### 2.1. Group I (M Group)

Patients in this group received sedation in the form of midazolam 3 mg i.v. added to fentanyl 0.5 ug/kg till reaching a satisfactory level of sedation. Supplementary dose of midazolam of 1 mg was given if the level of sedation reached was unsatisfactory.

### 2.2. Group II (P Group)

Patients in this group received sedation in the form of propofol 1 mg/kg i.v. added to fentanyl 0.5 ug/kg i.v till reaching a satisfactory level of sedation. If the level of sedation was not satisfactory, an additional dose of propofol 0.2 mg/kg will be given.

After adequate sedation of the patient, all patients received lidocaine spray 10% applied to the posterior pharynx for minimizing gag reflex followed by application of mouth piece. All the endoscopic maneuvers were performed in left lateral position with continuous monitoring by anesthesiologist till the end of the procedure and recovery of patients to the fully conscious level. All resuscitation equipment and medications were available.

All the following data were assessed including demographic data including age, sex, CTP class, body weight (BWT), type of endoscopic intervention, duration of endoscopy, time for complete recovery, and time for discharge. Additionally, time for complete recovery is the time elapsed between the end of endoscopy and the complete return of conscious level and time to discharge is the time elapsed between regaining conscious level and complete hospital discharge. The level of sedation using Ramsay sedation agitation score before induction of sedation (T0), 5 min after sedation (T1), 10 min after sedation (T2), and then every 30 min till complete recovery (T3, T4, and T5) was also assessed. Moreover, Endoscopist rating (easy or difficult endoscope) was recorded.

Hemodynamic parameters including oxygen saturation (Spo2), heart rate (HR), and mean arterial pressure (MAP) before induction (T0), 5 min after sedation (T1), 10 min after sedation (T2), and then every 15 min till complete recovery (T3, T4, T5, T6, T7, and T8) were monitored and incidence of side effects (bradycardia, hypotension, nausea and vomiting, shivering, diplopia, or hypoxia) was recorded.

Bradycardia is a decrease in HR less than 50 B/min, while hypotension is a decrease in mean arterial pressure by more than 20 mmHg. Hypoxia is a decrease in Spo2 less than 90%.

Bradycardia was managed by 0.3 mg i.v. atropine, while hypotension was managed by ephedrine 10 mg i.v. and ringer lactate 5 ml/kg. Hypoxia was managed by adequate airway management and oxygenation.

### 2.3. Statistical Analysis

The statistical differences between the studied groups were tested using unpaired *t*-test for parametric variables and chi-square test for nonparametric variables. The sedation score was analyzed with Wilcoxon test. Statistical tests were performed with SPSS (Version 23). *P* values < 0.05 were considered statistically significant.

## 3. Results

As regards patient characteristics of the two groups, there were no statistically significant differences between the studied groups regarding age, sex, CTP class, body weight, type of endoscopic intervention, duration of endoscopy, time for complete recovery, and time for discharge (Table 1).

Regarding sedation scores during the procedure, there were significant increases in sedation scores between T1, 2, 3, and 4 and baseline values in each group. However, there was no significant difference between T5 and baseline value in each group. In addition, there was no significant difference between groups in sedation scores (Table 2).

Considering endoscopist's rating, the rating was easy in 38% and 52% in M and P group, respectively. Moreover, the rating was difficult in 62% and 48% in M and P group, respectively. However, there were no significant differences in endoscopist's rating between both groups (Table 3).

Concerning side effects in studied groups, Hypotension was significantly more frequent in P group compared with M group, while there were no significant differences in frequency of bradycardia, nausea and vomiting, shivering, diplopia, or hypoxia between both groups (Table 4).

**Figure 1:**
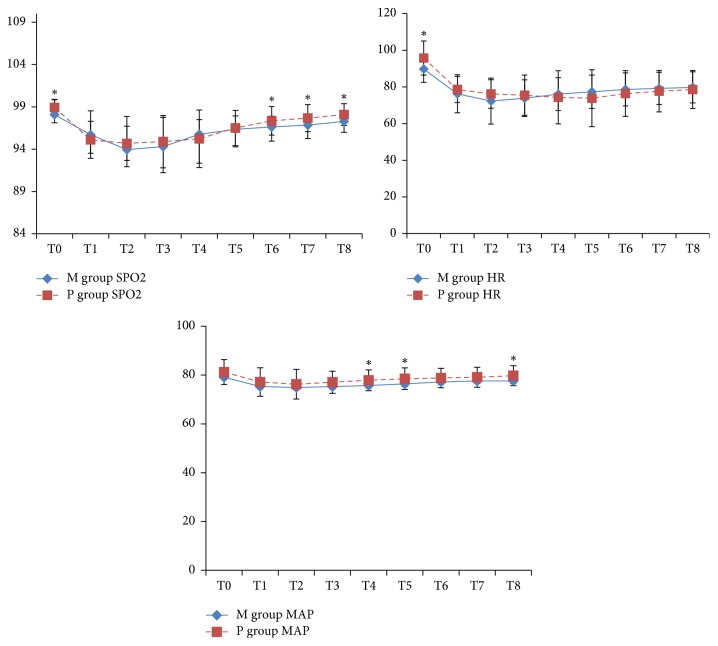
The changes in oxygen saturation (Spo2), heart rate (HR), and mean arterial pressure (MAP) in both studied groups. ^*∗*^*P* < 0.05 between the two groups.

Regarding the changes in oxygen saturation (Spo2), heart rate (HR) and mean arterial pressure (MAP) in both studied groups concerned with the Spo2 levels. As regards the Spo2 levels, P group showed significant increases at T0, T6, T7, and T8 when compared with M group. In addition, HR levels showed significant increases in P group at T0 only when compared with M group. Moreover, MAP showed significant increases in P group at T4, T5, and T8 when compared with M group (Figure 1).

## 4. Discussion

Patients with liver cirrhosis are commonly referred for upper gastrointestinal (GI) endoscopy for the screening and treatment of complications of portal hypertension, such as esophagogastric varices and portal hypertensive gastropathy [12].

The main goals of sedation in GI endoscopy are rapid onset of hypnosis, anxiolysis, analgesia, amnesia, cooperation to complete the procedure, and rapid recovery of consciousness [13, 14].

The aim of our randomized study was to assess the safety and efficacy of the use of propofol plus fentanyl versus midazolam plus fentanyl as sedatives for patients with advanced liver disease presented for gastrointestinal endoscopy.

There was no statistically significant difference between the studied groups regarding age, sex, weight, Child–Pugh classification score, and type and duration of endoscopic intervention, as some of these factors may affect the dose of the sedating drug or its related complications [15]. Also there was no significant difference regarding endoscopist's rating.

There was no statistical difference in time needed for complete recovery or time needed for discharging between two studied groups. In contrast to our results, Correia et al. 2011 [16] studied 200 cirrhotic patients and compared propofol and fentanyl with midazolam and fentanyl and found that sedation with propofol plus fentanyl was more efficacious with a shorter recovery time compared with midazolam plus fentanyl; also Seleem et al. 2014 [17] who studied 99 cirrhotic patients and divided them into three groups, midazolam plus fentanyl group, propofol plus fentanyl group, and Ketamine group, found that there was a high statistically significant difference among the studied groups with the shortest recovery time which was observed in propofol plus fentanyl group followed by midazolam plus fentanyl and Ketamine groups; also Poulos et al. 2013 [18] reported that the use of propofol plus fentanyl resulted in less time in the endoscopy unit, quicker recovery, and faster discharge than regimen using midazolam plus fentanyl, and Tsai et al. 2015 [19] who performed meta-analysis included five studies between 2003 and 2012, including 433 patients, and concluded that propofol provided a shorter time to sedation (weight mean difference: −2.76 min, 95% confidence interval: −3.00 to −2.51) and a shorter recovery time (weight mean difference −6.17 min, 95% confidence interval: −6.81 to −5.54) than midazolam did.

We also did not find statistical difference regarding side effects including nausea, vomiting, shivering, and diplopia. Also, heart rate and hypoxia among our patients groups showed no significant differences as we recorded 9 patients had bradycardia and 14 patients had hypoxia in M group while 14 patients had bradycardia and 12 patients had hypoxia in P group. On the other hand, there was a significant difference between the studied two groups regarding hypotension. As 4 patients in P group and only 1 patient in M group suffered from hypotension, the mechanisms by which midazolam causes hypotension was attributed to reduction in systematic vascular resistance and myocardial contractility [20, 21]; fentanyl causes hypotension due to a centrally mediated decrease in sympathetic tone as declared by Laubie et al., 1974 [22], while propofol causes hypotension by decreasing preload, cardiac output, and contractility [23, 24] or decreasing systemic vascular resistance [25].

This was in agreement with Seleem et al., 2014 [17] who found no significant difference in heart rate among two groups receiving propofol-fentanyl and midazolam-fentanyl; our results were in partial agreement with results of Correia et al., 2011 [16] who concluded that complications rates were similar in both groups, which was similar to our results except for hypotension. While Tsai et al. 2015 [19] recorded no intergroup difference regarding incidence of hypotension, bradycardia, or hypoxemia.

Similar to our results dos Santos et al., 2013 [26] who studied 200 patients (divided into two groups who received propofol-fentanyl or midazolam-fentanyl) concluded that there were significant differences between two studied groups regarding oxygen saturation as oxygen supplementation was required in 42% of the propofol-fentanyl group and 26% of the midazolam-fentanyl group.

Moerman et al., 2004 [27], who used the same dose of propofol (1 mg/kg) as our study recorded that patients received propofol plus fentanyl had serious hypotension, bradycardia, and hypoxemia in 14% out of 50 patients.

In contrast to our results Seleem et al. 2014 [17] found no significant difference in mean arterial pressure among studied groups. Also, Suh et al., 2014 [28] who performed upper gastrointestinal endoscopy under sedation with propofol along with careful monitoring in 20 patients with liver cirrhosis and 20 control subjects and found that there were neither respiratory depression nor clinically significant hypotension observed among cirrhotic patients.

On the other hand Barriga et al., 2008 [29] reported serious hypotension in 16 patients out of 480 patients who received midazolam plus fentanyl; also serious hypoxemia occurred in one patient and this may be attributed to their use of a higher dose of midazolam (0.1 mg/kg) than the dose we used in our study (3 mg) and higher number of patients included in their study.

Finally, we can conclude that the use of either propofol or midazolam in combination to fentanyl is effective in the sedation of patients with advanced liver diseases who presented for upper GIT endoscope with indifference between the two drugs as regarding level of sedation, difficulty of technique, duration of recovery or discharge, and incidence of complication. However, the risk of hypotension seems to be increased with use of propofol.

## Figures and Tables

**Table 1:** Patient characteristics of two groups.

	M group	P group	*P*
Age (year) mean ± SD	51.24 ± 5.80	49.94 ± 5.53	0.25

Gender			0.47
Male NO. (%)	28 (56%)	29.0 (58%)	
Female NO. (%)	22 (44%)	21.0 (42%)	

CTP class			0.34
B NO. (%)	31.00 (62%)	30.0 (60%)	
C NO. (%)	19.00 (38%)	20.0 (40%)	

Weight (kg) mean ± SD	73.02 ± 9.41	72.92 ± 8.88	0.96

Type of endoscopic intervention			0.22
Diagnostic NO. (%)	26 (52%)	25 (50%)	
Sclerotherapy NO. (%)	14 (28%)	17 (34%)	
Band ligation NO. (%)	10 (20%)	8 (16%)	

Duration of endoscope (min) mean ± SD	20.00 ± 5.80	20.90 ± 6.68	0.47

Time for complete recovery (min) mean ± SD	40.90 ± 11.90	36.60 ± 12.43	0.08

Time for discharge (min) mean ± SD	64.00 ± 14.98	61.80 ± 17.37	0.50

**Table 2:** Sedation scores during the procedure.

	M group (*n* = 50) Median (Minimum–maximum)	P group (*n* = 50) Median (Minimum–maximum)
T0	0 (0-1)	0 (0-1)
T1	4 (2–6)^*∗*^	4 (2–6)^*∗*^
T2	4 (3–6)^*∗*^	4 (3–6)^*∗*^
T3	4 (2–6)^*∗*^	4 (2–6)^*∗*^
T4	2 (1–5)^*∗*^	2 (1–5)^*∗*^
T5	0 (0-1)	0 (0-1)

^*∗*^ *P* < 0.05 compared with T0.

**Table 3:** Endoscopist's rating.

	M group	P group	*P*
Rating			0.22
Easy NO. (%)	19 (38%)	26 (52%)	
Difficult NO. (%)	31 (62%)	24 (48%)	

**Table 4:** Side effects in the studied groups.

	M group	P group	*P*
Bradycardia	9 (18%)	14 (28%)	0.67
Hypotension	1 (2%)	4 (8%)	0.001^*∗*^
Nausea and vomiting	30 (60%)	24 (48%)	0.13
Shivering	25 (50%)	23 (46%)	0.16
Diplopia	21 (42%)	19 (38%)	0.24
Hypoxia	14 (28%)	12 (24%)	0.64

^*∗*^ *P* < 0.05 (indicates significant difference between the studied groups). Bradycardia is a decrease in HR less than 50 B/min; hypotension is a decrease in mean arterial pressure by more than 20 mmHg.
